# Characteristics of Patients Accessing Outpatient Oncology Services Virtually and Predictors of Subsequent Unplanned Emergency Department Presentations in 78,323 Adults in Australia: Retrospective Cohort Study

**DOI:** 10.2196/87694

**Published:** 2026-04-30

**Authors:** Ramya Walsan, Reema Harrison, Johanna Westbrook, Ashfaq Chauhan, Michelle Moscova, Anita Vandyke, Elizabeth Manias, Tracey Webster, Natalie Taylor, Prince Peprah, Rebecca Mitchell

**Affiliations:** 1 Centre for Health Systems and Safety Research, Australian Institute of Health Innovation, Macquarie University, Level 6, 75 Talavera Road, North Ryde, New South Wales, 2109, Australia, 61 434862125; 2 Health ANSWERS, Shoalhaven, NSW, Australia; 3 Commerical & Capital Advisory, Change & Innovation, NSW Health Infrastructure, Sydney, NSW, Australia; 4 Monash Nursing and Midwifery, Faculty of Medicine, Nursing and Health Sciences, Monash University, Clayton, Victoria, Australia; 5 Transformation, Quality, & Safety Division, Clinical Leadership Effectiveness & Outcomes, Northern Health, Epping, Victoria, Australia; 6 Faculty of Medicine and Health, School of Population Health, UNSW Sydney, Sydney, New South Wales, Australia; 7 Centre for Healthcare Resilience and Implementation Science, Australian Institute of Health Innovation, Macquarie University, North Ryde, New South Wales, Australia

**Keywords:** cancer, virtual care, telehealth, unplanned health service use, emergency department visits

## Abstract

**Background:**

Virtual care has become increasingly integrated into oncology services since the COVID-19 pandemic, yet little is known about which patients use it most and how its use is associated with unplanned emergency care among people living with cancer.

**Objective:**

The study aims to identify sociodemographic and clinical predictors of virtual care use among patients accessing outpatient oncology services and quantify their association with unplanned emergency department (ED) visits.

**Methods:**

A retrospective cohort study was conducted using linked administrative health data for 78,323 adults with cancer who accessed outpatient oncology services in Victoria and Queensland, Australia, between January 2018 and December 2020, with a 1-year follow-up. Virtual care use and unplanned ED visits were categorized as none, low (1-3), or high (≥4), and analyzed using modified Poisson models with robust variance estimation, adjusted for sociodemographic and clinical factors.

**Results:**

Out of 78,323 patients, 37,706 (48.1%) did not use virtual care (only in-person), 24,196 (30.9%) had low use, and 16,421 (20.9%) were high users. Higher virtual care use was associated with rural (vs urban) residence (relative risk [RR] 1.23, 95% CI 1.19‐1.28), mental health disorders (vs none; RR 1.28, 95% CI 1.24‐1.33), Charlson comorbidities (vs none; RR 1.12, 95% CI 1.09‐1.28), and receiving care during the COVID-19 pandemic (vs nonpandemic period; RR 3.03, 95% CI 2.92‐3.15). In contrast, older age (≥75 y vs 18‐44 y; RR 0.78, 95% CI 0.74‐0.83) and being born overseas (vs Australia; RR 0.83, 95% CI 0.80‐0.86) were associated with lower virtual care use. High virtual care use (vs none) was associated with an increased risk of ≥4 unplanned ED visits (RR 2.64, 95% CI 2.52‐2.79).

**Conclusions:**

The use of virtual outpatient oncology services varied based on patients’ demographic and clinical characteristics. Higher virtual outpatient use was associated with increased unplanned ED presentations. Further research using causal analytic approaches is needed to clarify the relationship between virtual care and unplanned acute care use.

## Introduction

Cancer is a complex, often chronic condition requiring multidisciplinary, coordinated care from diagnosis through survivorship [1]. While cancer care was traditionally delivered in inpatient settings, advancements in treatment safety, increasing cost pressures, and a growing emphasis on patient-centered care have driven a shift toward outpatient and ambulatory models [2,3]. Virtual care, defined as the use of telephone, video, and other technological tools to deliver health care [4], was initially adopted in cancer services to improve access for rural and remote populations [4,5]. The COVID-19 pandemic, however, significantly accelerated its uptake across all health care settings [6]. This rapid integration yielded substantial benefits, including reduced travel burden [7], minimal disruption to work or education [8], and improved access for underserved populations [7].

Virtual care use in oncology has received relatively little attention, with existing studies primarily focusing on quality of life [9] and patient experiences [10]. To support postpandemic integration of virtual care, it is important to understand which patient groups are more or less likely to engage with virtual care, as well as its association with acute health service use, particularly unplanned emergency department (ED) visits. Unplanned ED presentations are a recognized indicator of gaps in outpatient care, often reflecting inadequate care coordination, limited access to timely support, and insufficient patient navigation services [11]. Although virtual care is expected to reduce unplanned service use by improving access and enabling earlier intervention, evidence on the nature of this association remains limited, particularly in Australia and among populations living with cancer [12,13].

A recent Australian study describing patients living with cancer found a higher likelihood of unplanned ED visits among those who used virtual services at least once, compared to those receiving in-person care [14]. However, it is not yet known whether these associations vary by the level of virtual care use, that is, whether higher levels of virtual care use are associated with higher unplanned ED use. This study aims to describe the sociodemographic and clinical characteristics associated with virtual care engagement and to examine the association between levels of virtual care use and unplanned ED visits among patients living with cancer.

## Methods

### Study Design and Setting

This retrospective cohort study was conducted using linked administrative health data from public nonadmitted patient or outpatient oncology services in Victoria and Queensland, Australia. The study included individuals aged 18 years or older who received nonadmitted patient or outpatient oncology services in Victoria and Queensland, Australia, between January 1, 2018, and December 31, 2020, and who had a confirmed cancer diagnosis in the Victorian or Queensland Cancer registries, respectively. These services deliver specialist cancer care within hospital-based outpatient clinics across the 2 states. Participants were followed for 1 year from their index outpatient encounter or were censored at death.

### Ethical Considerations

Ethical approval for the study was obtained from the Victorian Department of Health and the Department of Families, Fairness and Housing Human Research Ethics Committee (HREC/97793/DOH-2023-383794(v3)) and was approved by Queensland Health under Section 282 of the Public Health Act 2005 (Qld) s 282 (PHA 97793). As this study involved a retrospective analysis of routinely collected administrative data, a waiver of informed consent was granted by the approving ethics committees. Data linkage was conducted by the Centre for Victorian Data Linkage and the Queensland Statistical Services Branch, and researchers accessed only deidentified datasets within the Secure Unified Research Environment to ensure participant privacy and confidentiality. No compensation was provided to participants.

### Data Sources

Outpatient data from the Victorian Integrated Non-Admitted Health (VINAH) minimum dataset and the Queensland Health Non-Admitted Patient Data Collection (QHNAPDC) were linked to ED presentations, cancer registries, hospital admissions, and mortality datasets. VINAH and QHNAPDC capture all public outpatient services in Victoria and Queensland, including patient demographics, service dates, service types, contact streams, and delivery modes. Cancer-related data were obtained from the Victorian and Queensland Cancer Registries, which are population-based registries receiving notifications on cancer diagnoses, excluding nonmelanoma skin cancers. ED presentation data from the Victorian Emergency Minimum Dataset and Queensland Emergency Data Collection capture patient demographics, presentation details, and visit types. Comorbidity information was sourced from the Victorian Admitted Episodes Dataset and Queensland Hospital Admitted Patient Data Collection, which include admissions to both public and private hospitals classified using International Classification of Diseases, 10th Revision, Australian Modification (ICD-10-AM). Mortality data from the respective registries of births, deaths, and marriages provided the fact and date of death. Data linkage was performed by the Centre for Victorian Data Linkage and Queensland Statistical Services Branch.

### Case Inclusion Criteria

The study cohort included adults aged 18 years or older residing in Victoria or Queensland who received outpatient oncology services between January 1, 2018, and December 31, 2020, and had a cancer diagnosis (ICD-10-AM codes: C00–C43, C45–C96, D45, D46, D47.1, D47.3–D47.5) recorded in a cancer registry prior to or within 7 days of their first outpatient oncology encounter. Oncology service use was identified using contact stream ID 110 in the VINAH and Tier 2 clinic codes (oncology, medical oncology consultation, chemotherapy, and radiation oncology—consultation, simulation, and planning) in the QHNAPDC. Virtual care was defined as consultations delivered via telephone or video, based on the mode-of-delivery variable. To reduce selection bias, inclusion criteria were applied uniformly across both jurisdictions.

### Clinical Characteristics and Comorbidities

Cancer type was identified using primary site ICD-10-AM codes from cancer registry records, supplemented by inpatient data. The 4 most common cancer types in the dataset, such as breast, digestive (including colorectal), lung, and prostate, were analyzed individually, with all others combined (Table S1 in Multimedia Appendix 1). Comorbidities were determined from admission diagnoses, based on the 17 Charlson comorbidities, with malignant neoplasm excluded to prevent collinearity with cancer diagnosis, and were classified as present or absent. In addition, inpatient records were used to identify mental health disorders (ICD-10-AM: F00–F99) and tobacco use (ICD-10-AM: F17.0–F17.9, P04.2, T65.2, Z58.7, Z71.6, Z72.0, Z81.2, Z86.43). To account for prior outpatient health service use, individuals were categorized based on their preindex outpatient activity during the year preceding the study period (January 1 to December 31, 2017) as “low users,” defined as 0 to 3 outpatient encounters, while “high users” had ≥4 encounters [15].

### Sociodemographic Characteristics

Patients’ residential location in the outpatient data was classified using Statistical Area Level 2 (SA2) for Queensland and using postcode for Victoria, based on the Australian Statistical Geography Standard Remoteness classification. The 5 remoteness categories were collapsed into urban (major cities) and rural (ie, inner regional, outer regional, remote, and very remote areas). Socioeconomic disadvantage was assessed using the Index of Relative Socioeconomic Disadvantage, derived from the patient’s Statistical Area Level 2 (SA2)/postcode of residence, and classified into quintiles (Q1=most disadvantaged; Q5=least disadvantaged). Country of birth was categorized as Australian—or overseas—born from inpatient and outpatient records. Patients were classified as receiving treatment during the COVID-19 lockdown period if any portion of their 12-month follow-up period intersected with the defined COVID-19 lockdown period in both states (March 1, 2020-July 31, 2021).

### Study Outcomes

Outcomes were levels of virtual outpatient service use and unplanned ED visits during the 12-month follow-up. Based on the number of virtual consultations, patients were categorized into 3 groups: no virtual care use, low (1‐3 visits), and high (≥4 visits) use. Unplanned ED visits were identified using the visit type code classified as an “emergency presentation” and categorized as no, low (1‐3 visits), or high (≥4 visits) use [15].

### Data Management and Analysis

All analyses were performed using SAS 9.4 (SAS Institute Inc) within the Secure Unified Research Environment. Cohort characteristics and outcomes were summarized using descriptive statistics and Pearson chi-square tests. Modified Poisson regression models [16,17] with robust variance estimation were used to examine the association between patient characteristics and virtual care use and between virtual care use and unplanned ED visits within the same 12-month follow-up period. Among patients with both events, only 6% (4699/78,323) had an ED visit prior to their first virtual care encounter, indicating that, in the majority of cases, virtual care preceded ED presentation.

Separate fully adjusted models were fitted for each outcome and for each level, comparing low and high levels of each outcome with no use as the reference category [16]. Covariates were selected for their known association with service use and availability [18-20]. These included age group, gender, country of birth, Charlson comorbidities, remoteness, socioeconomic status, mental disorders, cancer type and morphology, COVID-19 lockdown period, and prior outpatient use.

Collinearity between covariates was assessed using pairwise correlation coefficients. Tobacco use was excluded due to strong collinearity with mental health disorders (*r*=0.90). Backward stepwise regression sequentially removed nonsignificant variables using a removal criterion of *P*>.05, and relevant 2-way interactions were tested. Analyses were conducted using complete cases and the proportion of missing data reported in the tables. A sensitivity analysis excluded unplanned ED visits with a COVID-19 diagnosis (ICD-10-AM code U07 as the principal or among the first 20 additional diagnoses). The results were reported as adjusted relative risks (RRs) with 95% CIs, with *P*≤.05 denoting significance.

## Results

### Characteristics of the Study Sample

The cohort included: 39,099 (49.9%) adults from Queensland and 39,224 (50.1%) from Victoria who accessed nonadmitted patient oncology services. Of these, 37,706 (48.1%) did not use virtual care during the 12-month follow-up, 24,196 (30.9%) had low virtual care use, and 16,421 had high (20.9%) virtual care use. High virtual care users had the highest proportion of unplanned ED visits, with 38.9% (n=4030) experiencing high ED use compared to 31.4% (n=3252) of low virtual care users and 29.8% (n=3091) of nonusers (Table 1, Table S2 in Multimedia Appendix 1).

**Table 1.** Unplanned ED^a^ visits among individuals aged 18 years or older who used virtual outpatient oncology services in Queensland and Victoria, Australia, from 2018 to 2021 (retrospective cohort study; N=78,323).

Characteristics	No unplanned ED use (n=39,512), n (%)	Low unplanned ED use (≤3 use/y) (n=28,438), n (%)	High unplanned ED use (≥4 use/y) (n=10,373), n (%)	*P* value
Virtual outpatient oncology use				
No use	22,680 (57.4)	11,935 (42)	3091 (29.8)	<.001
Low use (≤3 use/y)	11,440 (29)	9504 (33.4)	3252 (31.4)	
High use (≥4 use/y)	5392 (13.7)	6999 (24.6)	4030 (38.9)	
Age categories (y)				
18‐44	3177 (8)	2336 (8.2)	879 (8.5)	<.001
45‐64	12,529 (31.7)	8976 (31.6)	3309 (31.9)	
65‐74	11,416 (28.9)	7795 (27.4)	2872 (27.7)	
≥75	12,390 (31.4)	9331 (32.8)	3313 (31.9)	
Gender^b^				
Male	17,261 (43.7)	14,197 (49.9)	5654 (54.5)	.09
Female	22,249 (56.3)	14,241 (50.1)	4719 (45.5)	
Country of birth^c^				
Australia	26,888 (69.5)	19,544 (69.6)	7276 (71)	<.001
Other	11,796 (30.5)	8530 (30.4)	2968 (29)	
Rurality^d^				
Urban	25,969 (65.8)	17,141 (60.4)	5598 (54.1)	<.001
Rural	13,492 (34.2)	11,252 (39.6)	4760 (45.9)	
Charlson comorbidities (excluding malignant neoplasm)^e^				
No	20,595 (70.9)	13,156 (62.5)	4658 (53.3)	<.001
Yes	8453 (29.1)	7882 (37.5)	4077 (46.7)	
Other comorbidities				
Mental health disorder (yes)^e^	3620 (12.5)	4637 (22)	2892 (33.1)	<.001
Tobacco use (yes)^e^	13,210 (46.1)	11,239 (53.7)	5265 (60.6)	<.001
Socioeconomic status^f^				
Q1 (most disadvantaged)	7463 (18.9)	6718 (23.6)	2825 (27.2)	<.001
Q2	6461 (16.4)	5806 (20.4)	2347 (22.6)	
Q3	7741 (19.6)	5759 (20.3)	2035 (19.6)	
Q4	8054 (20.4)	5345 (18.8)	1704 (16.4)	
Q5 (most advantaged)	9784 (24.8)	4808 (16.9)	1461 (14.1)	
COVID-19 lockdown				
No	23,624 (59.8)	17,091 (60.1)	6319 (60.9)	.11
Yes	15,888 (40.2)	11,347 (39.9)	4054 (39.1)	
Cancer type				
Breast	10,540 (26.7)	5111 (18)	1248 (12)	<.001
Digestive organs including colorectal	5516 (14)	5950 (20.9)	2602 (25.1)	
Lung	2960 (7.5)	3975 (14)	1836 (17.7)	
Prostate	4643 (11.8)	2173 (7.6)	559 (5.4)	
Other cancers/unknown primary site/ill-defined	15,853 (40.1)	11,229 (39.5)	4128 (39.8)	
Cancer morphology				
Well or moderately differentiated	11,607 (29.4)	7664 (27)	2688 (25.9)	<.001
Poorly or undifferentiated	10,492 (26.6)	7273 (25.6)	2365 (22.8)	
Unspecified	17,413 (44.1)	13,501 (47.5)	5320 (51.3)	
Preindex outpatient use				
No use/low use	32,023 (81.1)	22,472 (79)	7798 (75.2)	<.001
High use (≥3/y)	7489 (18.9)	5966 (21)	2575 (24.8)	

a ED: emergency department.

b Data on gender were missing for 2 individuals and were not included in the analysis.

c Data on country of birth were missing for 1321 individuals and were not included in the analysis.

d Data on rurality were missing for 111 individuals and were not included in the analysis.

e Data on Charlson comorbidities, mental health disorders, and tobacco use were missing for 19,502 individuals and were not included in the analysis.

f Data on socioeconomic status were missing for 12 individuals and were not included in the analysis.

### Predictors of Virtual Outpatient Oncology Use

High users of virtual care had a slightly higher proportion of individuals aged 18 to 44 years (ie, n=1627, 9.9%) compared to low users (n=1864, 7.7%) and nonusers (n=1864, 7.7%) and a higher proportion residing in regional/rural areas (n=7163, 43.6% vs n=9827, 40.6% and n=12,514, 33.2% for low users and nonusers, respectively). Mental health disorders were slightly more common among high users (n=3090, 22.9%) than among low users (n=3087, 18.4%) and nonusers (n=4972, 17.4%). Among high users, there was a higher proportion of digestive cancers (n=3919, 23.9%) and a lower proportion of prostate cancer (n=840, 5.1%). A higher proportion of high virtual care users (n=9929, 60.5%) received care during the COVID-19 period (Table S2 in Multimedia Appendix 1).

Older individuals had a lower likelihood of high virtual care use compared to adults aged 18 to 44 years (eg, ≥75 y: RR 0.78; 95% CI 0.74‐0.83). Overseas-born individuals also had a lower likelihood of high virtual care use compared to adults born in Australia (RR 0.83; 95% CI 0.80‐0.86). Individuals from socioeconomically advantaged areas had a lower likelihood of virtual care use; for example, adults in the least disadvantaged areas had a 23% lower likelihood of high virtual care use compared with patients in the most disadvantaged areas (RR 0.77; 95% CI 0.74‐0.81).

Mental health disorders were associated with higher virtual care use (RR 1.28; 95% CI 1.24‐1.33), as were Charlson comorbidities (RR 1.12; 95% CI 1.09‐1.16), compared to individuals without these conditions. Compared to individuals with breast cancer, adults with digestive cancers (RR 1.24; 95% CI 1.19‐1.30) or lung cancer (RR 1.14; 95% CI 1.07‐1.20) had a higher rate of high virtual care use, while males with prostate cancer had a lower likelihood (RR 0.53; 95% CI 0.49‐0.57).

Virtual care uptake was higher during the COVID-19 period, with individuals more likely to have low use (RR 1.47; 95% CI 1.43‐1.51) and over 3 times more likely to have high use (RR 3.03; 95% CI 2.92‐3.15) compared to the non–COVID-19 period. Additionally, individuals with high preindex outpatient use were more likely to have high virtual care use (RR 1.38; 95% CI 1.31‐1.45) (Table S3 in Multimedia Appendix 1).

### Association Between Virtual Care Use and Unplanned ED Visit

The higher proportion of virtual care use was observed among adults with higher unplanned ED use. Among individuals with no ED visits, 57.4% (n=22,680) had not used virtual care, whereas among adults with high ED use, only 29.8% (n=3091) had not used virtual care and 38.9% (n=4030) had high virtual care use.

Among individuals with high unplanned ED use, 46.7% (n=4077) had Charlson comorbidities, 33.1% (n=2892) had mental health disorders, 60.6% (n=5265) reported tobacco use, 24.8% (n=2575) had high preindex outpatient use, and 45.9% (n=4760) resided in rural areas. Socioeconomic disadvantage was more common among high unplanned ED users, with 27.2% (n=2825) in the most disadvantaged quintile, compared with 23.6% (n=6718) among those with low ED use and 18.9% (n=7463) among those with no ED use. Lung (17.7%, n=1836) and digestive cancers (25.1%, n=2602) were more prevalent among high unplanned ED users, whereas breast (12.0%, n=1248) and prostate cancers (5.4%, n=559) were less common (Table 1).

Compared to nonvirtual care users, individuals with low virtual care use had 1.27 times the risk (95% CI 1.1.24‐1.31) and adults with high virtual care use had 2.64 times the risk (95% CI 2.52‐2.79) of high unplanned ED use. Older age was associated with lower risk, with individuals aged 75 years or older having a 35% lower risk of high unplanned ED use compared to adults aged 18 to 44 years (RR 0.65, 95% CI 0.60‐0.70). Females were less likely than males to have high unplanned ED use (RR 0.89, 95% CI 0.85‐0.93) (Figure 1 and Table S4 in Multimedia Appendix 1).

Compared to referent groups, individuals born outside Australia had a slightly higher risk of high ED use (RR 1.08; 95% CI 1.03‐1.13), as did rural residents (RR 1.15; 95% CI 1.10‐1.20), adults with Charlson comorbidities (RR 1.38; 95% CI 1.33‐1.44), mental health disorders (RR 1.64; 95% CI 1.57‐1.71), and high preindex outpatient users (RR 1.18; 95% CI 1.12‐1.24). Individuals from the least disadvantaged socioeconomic quintiles were less likely to experience unplanned ED use (RR 0.66; 95% CI 0.62‐0.70).

Receiving care during the COVID-19 period was associated with a lower risk of high unplanned ED use (RR 0.88; 95% CI 0.84‐0.92). Individuals with lung (RR 1.82; 95% CI 1.67‐1.98) or digestive (RR 1.74; 95% CI 1.61‐1.88) cancers had a higher risk of high unplanned ED use compared to adults with breast cancer (Figure 1 and Table S4 in Multimedia Appendix 1). No interaction effects were observed. Supplementary analysis excluding COVID-19-related encounters yielded similar results (Table S5 in Multimedia Appendix 1).

## Discussion

**Figure 1.**
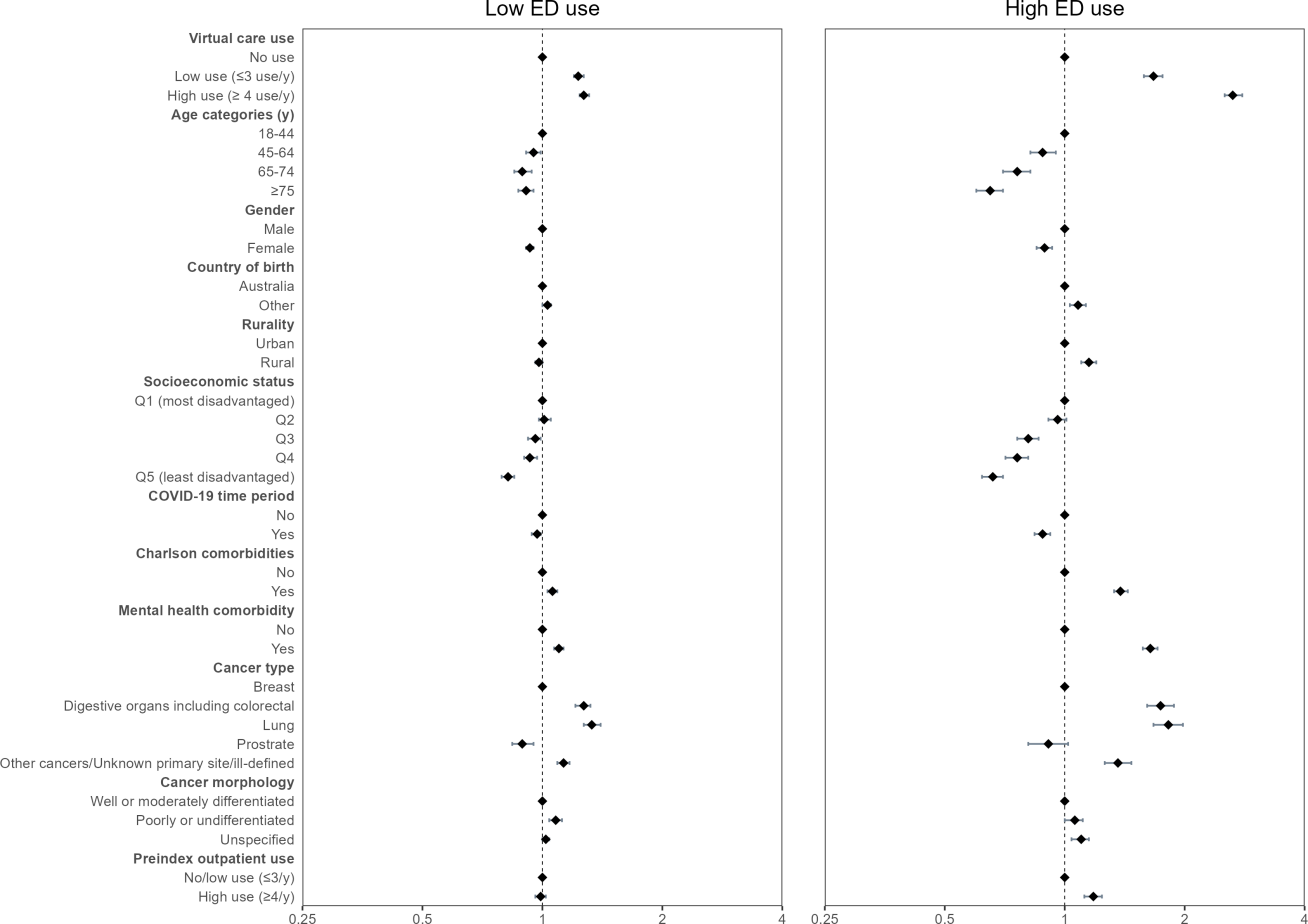
Virtual outpatient oncology use and unplanned ED visits among individuals aged ≥18 years who used outpatient oncology services in Queensland and Victoria, Australia, from 2018 to 2021 (retrospective cohort study; N=78,323). ED: emergency department.

### Main Findings

This study investigated factors associated with virtual care use and its association with unplanned ED visits among patients with cancer receiving outpatient care in Victoria and Queensland. Virtual care was more frequently used by younger adults, individuals residing in rural areas, and those with mental health conditions or a greater burden of Charlson comorbidities. Use was also higher during the COVID-19 lockdown period. In contrast, older adults and individuals born overseas were less likely to use virtual outpatient services. Notably, higher levels of virtual care use were associated with increased unplanned ED presentations.

### Comparison With Existing Literature

The observed disparities in virtual care use among older adults and individuals born overseas align with previous research [21,22] and could reflect barriers, such as digital literacy, limited access to devices or reliable internet, and lower confidence in using technology for health purposes [23]. Similarly, individuals born overseas may encounter language barriers, cultural preferences for in-person care, and reduced access to digital services [24]. Adults with a higher comorbidity burden, including mental health disorders, were more frequent users of virtual care, potentially due to an increased need for regular monitoring, symptom management, and multidisciplinary input [25]. Additionally, virtual care uptake was higher during the COVID-19 lockdown period, as health services shifted online to maintain continuity and minimize infection risk. Cancer type was also associated with virtual care use, with higher use observed among individuals with digestive or lung cancers compared to adults with breast cancer. This may reflect greater clinical complexity and a higher need for ongoing monitoring and management in these cancers [26,27].

Higher virtual care use was associated with a greater likelihood of unplanned ED presentations in this study. Similar associations have been reported in prior studies [28,29], whereas others have reported no difference or reduced use [30,31]. It is important to note that these associations may reflect underlying differences in patient characteristics, and a causal relationship with the mode of care cannot be inferred. A higher likelihood of unplanned ED visits may reflect greater clinical complexity among virtual care users, who were more likely to have comorbidities and high prior service use, all markers of greater care needs. In oncology, where rapid deterioration is common, virtual consultations may prompt early symptom detection and precautionary ED referrals for evaluation when in-person assessment is not feasible (eg, directing a patient reporting chest pain or fever straight to the ED). Notably, only 2.6% of ED visits in our sample were coded under ICD-10-AM Z00–Z13 (examination and investigation), suggesting that most ED presentations were not investigation-only encounters; however, these codes are assigned after clinical assessment and do not capture referral rationale. Alternatively, this observed association may indicate a more proactive care-seeking behavior among virtual care users or limitations in virtual care’s ability to manage complex needs.

Older adults with cancer had a lower likelihood of unplanned ED visits, consistent with prior research among cancer survivors suggesting decreased ED use with age [18], possibly due to preferences for conservative care or greater engagement with other services or death [32]. Women had lower odds of unplanned ED visits, aligning with higher acute care use reported among male patients with cancer [19]. Patients in the socioeconomically least disadvantaged areas were less likely to present to the ED for an unplanned visit, potentially indicating better access to timely, coordinated care [33,34]. In contrast, individuals born overseas and adults living in rural areas had higher odds of unplanned ED use, which may be associated with systemic barriers, including limited access to primary or specialist care [35], language barriers [36], and longer travel times [37]. These findings align with literature on greater ED reliance in rural and underserved populations [35,38]. The COVID-19 lockdown period was associated with reduced unplanned ED presentations, reflecting international trends during the pandemic [39]. This decrease may be related to public health messaging, infection fears, and system-level measures discouraging hospital attendance [40].

Comorbidities, including mental health disorders, were associated with unplanned ED use, consistent with their known impact on clinical deterioration and crisis-driven health care utilization [25]. Patients with digestive and lung cancers had the highest likelihood of unplanned ED presentations, likely due to acute complications and rapid disease progression [26,27,41]. Increased preindex engagement with outpatient services was also associated with higher ED use, suggesting that prior care intensity may signal greater underlying need or a tendency toward more proactive care-seeking.

While conducted in a health system with relatively higher digital capacity, these findings are relevant to other settings, particularly those with larger digital divides or constrained oncology resources, where inequities in virtual care access may be amplified. Targeted interventions, such as digital literacy support for older adults and multilingual telehealth services for migrant populations, may help promote equitable access [42,43]. Further research is needed to better understand the content of virtual encounters, subsequent acute care pathways, and the extent to which observed associations reflect causal effects versus underlying patient complexity.

### Strengths and Limitations

A key strength of this study is the use of large, population-based linked administrative datasets from both Victoria and Queensland, providing valuable insights into virtual oncology service use and unplanned ED presentations among patients living with cancer. However, its cross-sectional design limits interpretation to associations and precludes causal inference. Virtual care use and outcomes were measured within the same follow-up window, and despite virtual care preceding ED presentation for most patients, residual confounding is possible. Information on cancer stages and treatment types was not available, limiting the ability to account for treatment intensity, eligibility for virtual care use, and disease severity. Comorbidities were limited to hospital-recorded diagnoses, likely capturing only the most severe cases and resulting in underenumeration. However, the 1-year lookback period likely reduced but did not eliminate this limitation. Residual confounding may persist, as adults with more severe symptoms could preferentially choose virtual care, partly explaining its association with unplanned ED use. Virtual care use was classified using absolute visit counts, which may mask heterogeneity in clinical patterns across categories, particularly among higher-use patients. The use of a complete case approach may have introduced selection bias if missing data were not random. Preferred language was not included in the analysis due to substantial missing data (47%, n=36,812) and strong collinearity with country of birth. The provision of treatment during the COVID-19 pandemic further complicated interpretation, given service disruptions, rapid telehealth expansion, and varying digital readiness. However, a COVID-19 lockdown time period was included as a covariate in the analysis to distinguish nonpandemic and pandemic phases of care delivery. A stratified prepandemic and postpandemic analysis was not feasible due to low virtual care provision in the pre-COVID period. Finally, the content, quality, the modality of virtual encounters, or administrative data were not able to be validated.

### Conclusion

This study highlighted disparities in virtual oncology use, with uptake highest among younger patients, rural residents, and adults with comorbidities, and lowest among older adults and individuals born overseas. To promote equity, policymakers and service planners should work to identify the factors driving these disparities and ensure appropriate use of virtual care. Although virtual care enhances timely access, its association with higher unplanned ED presentations likely reflects greater clinical complexity and differences in care pathways. Further research, including studies using causal analytic approaches, is needed to better understand how virtual consultations are conducted, how they are integrated into broader care pathways, and how these factors influence acute care use.

## Supplementary material

10.2196/87694 Multimedia Appendix 1 Cancer type classification, patient characteristics, predictors of virtual outpatient oncology use, and its association with unplanned emergency department presentations.

## Abbreviations

ED: emergency department
ICD-10-AM: International Classification of Diseases, 10th Revision, Australian Modification
QHNAPDC: Queensland Health Non-Admitted Patient Data Collection
RR: relative risk
VINAH: Victorian Integrated Non-Admitted Health

## References

[1] Phillips JL, Currow DC (2010). Cancer as a chronic disease. Collegian.

[2] Wu IQ, Lim F, Koh LP (2022). The Comprehensive Cancer Center: Development, Integration, and Implementation.

[3] Williamson TS (2008). The shift of oncology inpatient care to outpatient care: the challenge of retaining expert oncology nurses. Clin J Oncol Nurs.

[4] Harrison R, Manias E (2022). How safe is virtual healthcare?. Int J Qual Health Care.

[5] Harkey LC, Jung SM, Newton ER, Patterson A (2020). Patient satisfaction with telehealth in rural settings: a systematic review.. Int J Telerehabil.

[6] Seivert S, Badowski ME (2021). The rise of telemedicine: lessons from a global pandemic. EMJ Innov.

[7] Jue JS, Spector SA, Spector SA (2017). Telemedicine broadening access to care for complex cases. J Surg Res.

[8] Rodin D, Lovas M, Berlin A (2020). The reality of virtual care: implications for cancer care beyond the pandemic. Healthc (Amst).

[9] Larson JL, Rosen AB, Wilson FA (2018). The effect of telehealth interventions on quality of life of cancer patients: a systematic review and meta-analysis. Telemed J E Health.

[10] Cox A, Lucas G, Marcu A (2017). Cancer survivors’ experience with telehealth: a systematic review and thematic synthesis. J Med Internet Res.

[11] Alishahi Tabriz A, Turner K, Hong YR, Gheytasvand S, Powers BD, Elston Lafata J (2023). Trends and characteristics of potentially preventable emergency department visits among patients with cancer in the US. JAMA Netw Open.

[12] Sina M, Mitchell R, Walsan R (2025). Using virtual models of care for chronic disease management in outpatient services: a systematic review of quality of care outcomes. Telemed J E Health.

[13] Shaffer KM, Turner KL, Siwik C (2023). Digital health and telehealth in cancer care: a scoping review of reviews. Lancet Digit Health.

[14] Walsan R, Harrison R, Braithwaite J Predictors of Unplanned Emergency Department Visits and Hospitalization Among Adults Accessing Virtual Outpatient Oncology Services in Queensland, Australia. Telemedicine and e-Health.

[15] Soril LJJ, Leggett LE, Lorenzetti DL, Noseworthy TW, Clement FM (2016). Characteristics of frequent users of the emergency department in the general adult population: a systematic review of international healthcare systems. Health Policy.

[16] Camey SA, Torman VBL, Hirakata VN, Cortes RX, Vigo A (2014). Bias of using odds ratio estimates in multinomial logistic regressions to estimate relative risk or prevalence ratio and alternatives. Cad Saude Publica.

[17] Iglesias-Rios L, Harlow SD, Reed BD (2015). Depression and posttraumatic stress disorder among women with vulvodynia: evidence from the population-based woman to woman health study. J Womens Health (Larchmt).

[18] Abdel-Rahman O (2021). Gender, socioeconomic status and emergency department visits among cancer survivors in the USA: a population-based study. J Comp Eff Res.

[19] Lash RS, Bell JF, Reed SC (2017). A systematic review of emergency department use among cancer patients. Cancer Nurs.

[20] Handley NR, Schuchter LM, Bekelman JE (2018). Best practices for reducing unplanned acute care for patients with cancer. J Oncol Pract.

[21] Mistry SK, Shaw M, Raffan F (2022). Inequity in access and delivery of virtual care interventions: a scoping review. Int J Environ Res Public Health.

[22] Abdel-Rahman O (2021). Patient-related barriers to some virtual healthcare services among cancer patients in the USA: a population-based study. J Comp Eff Res.

[23] Hall Dykgraaf S, Desborough J, Sturgiss E, Parkinson A, Dut GM, Kidd M (2022). Older people, the digital divide and use of telehealth during the COVID-19 pandemic. Aust J Gen Pract.

[24] Mahumud RA, Shahjalal M, Dahal PK (2025). Emerging burden of post-cancer therapy complications on unplanned hospitalisation and costs among Australian cancer patients: a retrospective cohort study over 14 years. Sci Rep.

[25] Sarfati D, Koczwara B, Jackson C (2016). The impact of comorbidity on cancer and its treatment. CA Cancer J Clin.

[26] Grewal K, Calzavara A, McLeod SL (2024). Emergency department use before cancer diagnosis in Ontario, Canada: a population-based study. CMAJ.

[27] Thompson CA, Sheridan P, Metwally E (2024). Emergency department involvement in the diagnosis of cancer among older adults: a SEER-Medicare study. JNCI Cancer Spectr.

[28] Hatef E, Lans D, Bandeian S, Lasser EC, Goldsack J, Weiner JP (2022). Outcomes of in-person and telehealth ambulatory encounters during COVID-19 within a large commercially insured cohort. JAMA Netw Open.

[29] Waseem N, Boulanger M, Yanek LR, Feliciano JL (2022). Disparities in telemedicine success and their association with adverse outcomes in patients with thoracic cancer during the COVID-19 pandemic. JAMA Netw Open.

[30] Shah VV, Villaflores CW, Chuong LH (2022). Association between in-person vs telehealth follow-up and rates of repeated hospital visits among patients seen in the emergency department. JAMA Netw Open.

[31] Bange EM, Li Y, Kumar P (2024). The association between telemedicine, advance care planning, and unplanned hospitalizations among high-risk patients with cancer. Cancer.

[32] Tolppanen AM, Lamminmäki A, Kataja V, Tyynelä-Korhonen K (2024). Specialized palliative outpatient clinic care involvement associated with decreased end-of-life hospital costs in cancer patients, a single center study. BMC Palliat Care.

[33] Walsan R, Mayne DJ, Feng X, Pai N, Bonney A (2019). Examining the association between neighbourhood socioeconomic disadvantage and type 2 diabetes comorbidity in serious mental illness. Int J Environ Res Public Health.

[34] Sheringham J, Asaria M, Barratt H, Raine R, Cookson R (2017). Are some areas more equal than others? Socioeconomic inequality in potentially avoidable emergency hospital admissions within English local authority areas. J Health Serv Res Policy.

[35] Stephens AS, Dinh MM, Kinsman L (2023). Patterns of emergency department use in rural and metropolitan New South Wales by socioeconomic status: a population-based study. Emerg Med Australas.

[36] Chu JN, Wong J, Bardach NS (2024). Association between language discordance and unplanned hospital readmissions or emergency department revisits: a systematic review and meta-analysis. BMJ Qual Saf.

[37] Cerni J, Rhee J, Hosseinzadeh H (2020). End-of-life cancer care resource utilisation in rural versus urban settings: a systematic review. Int J Environ Res Public Health.

[38] Credé SH, Such E, Mason S (2018). International migrants’ use of emergency departments in Europe compared with non-migrants’ use: a systematic review. Eur J Public Health.

[39] Patt D, Gordan L, Diaz M (2020). Impact of COVID-19 on cancer care: how the pandemic is delaying cancer diagnosis and treatment for American seniors. JCO Clin Cancer Inform.

[40] Lange SJ, Ritchey MD, Goodman AB (2020). Potential indirect effects of the COVID-19 pandemic on use of emergency departments for acute life-threatening conditions - United States, January-May 2020. Am J Transplant.

[41] Galdas PM, Cheater F, Marshall P (2005). Men and health help-seeking behaviour: literature review. J Adv Nurs.

[42] Jung J, You J, Kim D (2025). Effective but sustainable? A case of a digital literacy program for older adults. Educ Inf Technol.

[43] Gallegos-Rejas VM, De Guzman KR, Kelly JT, Smith AC, Thomas EE (2025). Strategies to improve telehealth access for culturally and linguistically diverse communities: a systematic review. J Public Health (Oxf).

