# Supplementary material for: Characteristics of Patients Accessing Outpatient Oncology Services Virtually and Predictors of Subsequent Unplanned Emergency Department Presentations in 78,323 Adults in Australia: Retrospective Cohort Study
Source: J Med Internet Res. 2026 Apr 30;28:e87694. doi: 10.2196/87694 (PMC13132016; doi:10.2196/87694)
Supplement: Multimedia Appendix 1 [file jmir-v28-e87694-s001.docx]

**Supplementary Table 1: Identification and Categorisation of Cancer types**

| **Cancer type** | **ICD-10-AM** |
| --- | --- |
| Breast | C50 |
| Digestive organs, including colorectal | C15-C17, C18-C20, C21-C26, |
| Lung | C33-C34 |
| Prostrate | C61 |
| Other cancers/ Unknown primary site/ill defined | C43, C44, C00-C14, C30-C32, C45-C49, C51-C55, C57.1-C57.9, C58.1-C58.9, C56, C57.0, C58.0, D359, C64, C67, C71, C72.8, C72.9, C81-C86, C88, C90-C96, D45, D46, C47.1, D47.3-D47.5, C80, C30-C32, C37-C39, C40-C41, C60, C62 -C63, C65-C66, C68, C69, C70, C72.0, C72.7, C73, C74, C75.0, C75.1-C75.3, C75.4-C75.9, C76, C60, C62-C63, C77-C78, C79 |

**Supplementary Table 2. Characteristics of Adults Using Virtual Non-Admitted Outpatient Oncology Services in Queensland and Victoria, Australia, 2018–2021 (retrospective cohort study, n = 78,323)**

| Characteristics | No virtual care use (n=37,706) | | Low virtual care use  [≤3 use/year]  (n=24,196) | | High virtual care use  [≥4 use/year]  (n=16,421) | | P value |
| --- | --- | --- | --- | --- | --- | --- | --- |
|  | **n** | **%** | **n** | **%** | **n** | **%** |  |
| Age categories | | | | | | | |
| 18 – 44 years | 2,901 | 7.7 | 1,864 | 7.7 | 1,627 | 9.9 | <0.001 |
| 45 – 64 years | 11,195 | 29.7 | 8,145 | 33.7 | 5,474 | 33.3 |  |
| 65 – 74 years | 10,537 | 28.0 | 7,081 | 29.3 | 4,465 | 27.2 |  |
| ≥75 years | 13,073 | 34.7 | 7,106 | 29.4 | 4,855 | 29.6 |  |
| Gender ^a^ | | | | | | | |
| Male | 17,709 | 47.0 | 11,632 | 48.1 | 7,771 | 47.3 | 0.093 |
| Female | 19,996 | 53.0 | 12,563 | 51.9 | 8,650 | 52.7 |  |
| Country of birth ^b^ | | | | | | | |
| Australia | 25,194 | 68.3 | 16,894 | 70.7 | 11620 | 71.7 | <0.001 |
| Other | 11,710 | 31.7 | 6,999 | 29.3 | 4,585 | 28.3 |  |
| Rurality ^c^ |  |  |  |  |  |  |  |
| Urban | 25,149 | 66.7 | 14,337 | 59.3 | 9,222 | 56.2 | <0.001 |
| Rural | 12,514 | 33.2 | 9,827 | 40.6 | 7,163 | 43.6 |  |
| Charlson comorbidities (excluding malignancy) ^d^ | | | | | | | |
| No | 18,481 | 64.6 | 11,390 | 67.9 | 8,538 | 63.4 | <0.001 |
| Yes | 10,115 | 35.3 | 5,375 | 32.1 | 4,922 | 36.6 |  |
| Other comorbidities | | | | | | | |
| Mental health disorder (yes) ^d^ | 4,972 | 17.4 | 3,087 | 18.4 | 3,090 | 22.9 | <0.001 |
| Tobacco use (Yes) ^d^ | 14,204 | 50.2 | 8,352 | 50.1 | 7,158 | 53.8 | <0.001 |
| Alcohol misuse and dependence (Yes) ^d^ | 591 | 2.1 | 338 | 2.0 | 296 | 2.2 | 0.493 |
| Drug-related dependence ^d^ | 202 | 0.7 | 130 | 0.8 | 131 | 1.0 | 0.148 |
| Socio economic status ^e^ | | | | | | | |
| Q1 (Most disadvantaged) | 7.499 | 19.9 | 5,883 | 24.3 | 3,624 | 22.1 | <0.001 |
| Q2 | 6,095 | 16.2 | 4,968 | 20.5 | 3,551 | 21.6 |  |
| Q3 | 7,355 | 19.5 | 4,725 | 19.5 | 3,455 | 21.0 |  |
| Q4 | 7,571 | 20.1 | 4,423 | 18.3 | 3,109 | 18.9 |  |
| Q5 (Most advantaged) | 9,180 | 24.4 | 4,193 | 17.3 | 2,680 | 16.3 |  |
| COVID-19 lockdown | | | | | | | |
| No | 28,096 | 74.5 | 12,446 | 51.4 | 6,492 | 39.5 | <0.001 |
| Yes | 9,610 | 25.4 | 11,750 | 48.6 | 9,929 | 60.5 |  |
| Cancer type | | | | | | | |
| Breast | 8,349 | 22.1 | 5,228 | 21.6 | 3,322 | 20.2 | <0.001 |
| Digestive organs including colorectal | 5,989 | 15.9 | 4,160 | 17.2 | 3,919 | 23.9 |  |
| Lung | 3,554 | 9.4 | 2,915 | 12.1 | 2,302 | 14.0 |  |
| Prostrate | 3,868 | 10.3 | 2,667 | 11.0 | 840 | 5.1 |  |
| Other cancers/ unknown primary site/ill defined | 15,946 | 42.3 | 9,226 | 38.1 | 6,038 | 36.8 |  |
| Cancer morphology | | | | | | | |
| Well or moderately differentiated | 10,436 | 27.7 | 6,828 | 28.2 | 4,695 | 28.6 | <0.001 |
| Poorly or undifferentiated | 9,401 | 24.9 | 6,505 | 26.9 | 4,224 | 25.7 |  |
| Unspecified | 17,869 | 47.4 | 10,863 | 44.9 | 7,502 | 45.7 |  |
| Pre-index outpatient use | | | | | | | |
| No use /low use | 29,006 | 76.9 | 19,662 | 81.3 | 13,625 | 83.0 | <0.001 |
| High use (≥3/year) | 8,700 | 23.1 | 4,534 | 18.7 | 2,796 | 17.0 |  |

^a Information on gender was not available for 2 individual was not included in the analysis.^

^b Information on country of birth was missing for 1321 individuals was not included in the analysis.^

^c Data on rurality was missing for 111 individuals was not included in the analysis^

^d Data on Charlson comorbidities, mental health disorders, tobacco use was missing for 19,502 individuals and was excluded from the analysis.^

^e Data on socioeconomic status was missing for 12 individuals and was not included in the analysis.^

**Supplementary Table 3: Predictors of virtual outpatient oncology use among individuals ≥18 years who used outpatient oncology services in Queensland and Victoria, Australia 2018 – 2021(retrospective cohort study, n = 78,323)**

| **Characteristics** | **Low virtual NAP use [≤3 use/year]** | **High Virtual NAP use**  **[≥4 use/year]** | |
| --- | --- | --- | --- |
|  | RR (95% CI) | RR (95% CI) | P value |
| **Age Group** | | | |
| 18 – 44 years | 1 | 1 | <0.001 |
| 45 – 64 years | 1.10 (1.00 – 1.17) | 0.81 (0.77 – 0.85) |  |
| 65 – 74 years | 1.07 (1.00 – 1.13) | 0.76 (0.72 – 0.81) |  |
| ≥75 years | 0.90 (0.85 – 1.06) | 0.78 (0.74 – 0.83) |  |
| **Country of birth** |  |  |  |
| Australia | 1 | 1 | <0.001 |
| Other | 0.98 (0.95 – 1.01) | 0.83 (0.80 – 0.86) |  |
| **Rurality** | | | |
| Urban | 1 | 1 | <0.001 |
| Rural | 1.04 (1.01 – 1.07) | 1.23 (1.19 – 1.28) |  |
| **Charlson comorbidity (excluding malignancy)** | | | |
| No | 1 | 1 | <0.001 |
| Yes | 0.92 (0.89 – 0.94) | 1.12 (1.09 – 1.16) |  |
| **Mental health disorders** | | | |
| No | 1 | 1 | <0.001 |
| Yes | 1.02 (0.98 – 1.05) | 1.28 (1.24 – 1.33) |  |
| **Socio economic status** | | | |
| Q1 (Most disadvantaged) | 1 | 1 | <0.001 |
| Q2 | 0.96 (0.93 – 1.00) | 1.14 (1.09 – 1.19) |  |
| Q3 | 0.87 (0.84 – 0.91) | 1.01 (0.97 – 1.06) |  |
| Q4 | 0.84 (0.81 – 0.88) | 0.95 (0.91 – 1.00) |  |
| Q5 (Least disadvantaged) | 0.75 (0.72 – 0.78) | 0.77 (0.74 – 0.81) |  |
| **COVID-19 lockdown** | | | |
| No | 1 | 1 | <0.001 |
| Yes | 1.47 (1.43 – 1.51) | 3.03 (2.92 – 3.15) |  |
| **Cancer type** | | | |
| Breast | 1 | 1 | <0.001 |
| Digestive organs including colorectal | 1.05 (1.00 – 1.09) | 1.24 (1.19 – 1.30) |  |
| Lung | 1.17 (1.11 – 1.24) | 1.14 (1.07 – 1.20) |  |
| Prostrate | 1.31 (1.25 – 1.38) | 0.53 (0.49 – 0.57) |  |
| Other cancers/ unknown primary site/ill defined | 1.09 (1.05 – 1.14) | 0.86 (0.82 – 0.90) |  |
| **Cancer morphology** | | | |
| Well or moderately differentiated | 1 | 1 | <0.001 |
| Poorly or undifferentiated | 1.00 (0.97 – 1.04) | 1.06 (1.01 – 1.10) |  |
| Unspecified | 0.93 (0.90 – 0.96) | 1.06 (1.03 – 1.10) |  |
| **Pre-index outpatient use** | | | |
| No use /low use | 1 | 1 | <0.001 |
| High use (≥3/year) | 1.12(1.08 – 1.17) | 1.38 (1.31 – 1.45) |  |

**Supplementary Table 4: Association between virtual outpatient oncology use and unplanned ED visits use among individuals ≥18 years who used outpatient oncology services in Queensland and Victoria, Australia, 2018 – 2021(retrospective cohort study, n = 78,323)**

| **Characteristics** | **Low unplanned ED use**  **[≤3 use/year]** | **High unplanned ED use**  **[≥4 use/year]** | |
| --- | --- | --- | --- |
|  | RR (95 % CI) | RR (95 % CI) | P value |
| **Virtual outpatient oncology use** | | | |
| No use | 1 |  | <0.001 |
| Low use (≤3 use/year) | 1.23 (1.20 – 1.27) | 1.67 (1.58 – 1.76) |  |
| High use (≥ 4 use/year) | 1.27 (1.24 – 1.31) | 2.64 (2.52 – 2.79) |  |
| **Age group** | | | |
| 18 – 44 years | 1 | 1 | <0.001 |
| 45 – 64 years | 0.95 (0.91 – 0.99) | 0.88 (0.82 – 0.95) |  |
| 65 – 74 years | 0.89 (0.85 – 0.94) | 0.76 (0.70 – 0.82) |  |
| ≥75 years | 0.91 (0.87 – 0.95) | 0.65 (0.60 – 0.70) |  |
| **Sex** | | | |
| Male | 1 | 1 | <0.001 |
| Female | 0.93 (0.91 – 0.95) | 0.89 (0.85 – 0.93) |  |
| **Country of birth** |  |  |  |
| Australia | 1 | 1 | <0.001 |
| Other | 1.03 (1.00 – 1.05) | 1.08 (1.03 – 1.13) |  |
| **Rurality** | | | |
| Urban | 1 | 1 | <0.001 |
| Rural | 0.98 (0.96 – 1.01) | 1.15 (1.10 – 1.20) |  |
| **Charlson comorbidity (excluding malignancy)** | | | |
| No | 1 | 1 | <0.001 |
| Yes | 1.06 (1.03 – 1.09) | 1.38 (1.33 – 1.44) |  |
| **Mental health disorders** | | | |
| No | 1 | 1 | <0.001 |
| Yes | 1.10 (1.07 – 1.13) | 1.64 (1.57 – 1.71) |  |
| **Socio economic status** | | | |
| Q1 (Most disadvantaged) | 1 | 1 | <0.001 |
| Q2 | 1.01 (0.98 – 1.05) | 0.96 (0.91 – 1.01) |  |
| Q3 | 0.96 (0.92– 0.99) | 0.81 (0.76 – 0.86) |  |
| Q4 | 0.93 (0.90 – 0.97) | 0.76 (0.71 – 0.81) |  |
| Q5 (Most advantaged) | 0.82 (0.79 – 0.85) | 0.66 (0.62 – 0.70) |  |
| **COVID-19 lockdown** | | | |
| No | 1 | 1 | <0.001 |
| Yes | 0.97 (0.94 – 0.99) | 0.88 (0.84 – 0.92) |  |
| **Cancer type** | | | |
| Breast | 1 | 1 | <0.001 |
| Digestive organs including colorectal | 1.27 (1.21 – 1.32) | 1.74 (1.61 – 1.88) |  |
| Lung | 1.33 (1.27 – 1.40) | 1.82 (1.67 – 1.98) |  |
| Prostrate | 0.89 (0.84 – 0.95) | 0.91 (0.81 – 1.02) |  |
| Other cancers/ unknown primary site/ill defined | 1.13 (1.09 – 1.17) | 1.36 (1.26 – 1.47) |  |
| **Cancer morphology** | | | |
| Well or moderately differentiated | 1 | 1 | <0.001 |
| Poorly or undifferentiated | 1.08 (1.04 – 1.12) | 1.06 (1.00 – 1.11) |  |
| Unspecified | 1.02 (1.00 – 1.04) | 1.10 (1.04 – 1.15) |  |
| **Pre-index outpatient use** | | | |
| No use /low use | 1 | 1 | <0.001 |
| High use (≥3/year) | 0.99 (0.96 – 1.01) | 1.18 (1.12 – 1.24) |  |

**Supplementary Table 5: Association between virtual outpatient oncology use and unplanned ED visits (excluding unplanned ED visits with COVID-19 diagnosis) among individuals ≥18 years who used outpatient oncology services in Queensland and Victoria, Australia, 2018 – 2021(retrospective cohort study, n = 78,323)**

| **Characteristics** | **Low unplanned ED use**  **[≤3 use/year]** | **High unplanned ED use**  **[≥4 use/year]** | |
| --- | --- | --- | --- |
|  | RR (95 % CI) | RR (95 % CI) | P value |
| **Virtual outpatient oncology use** | | | |
| No use | 1 |  | <0.001 |
| Low use (≤3 use/year) | 1.19 (1.15 – 1.24) | 1.43 (1.35 – 1.51) |  |
| High use (≥ 4 use/year) | 1.22 (1.19 – 1.26) | 2.27 (2.15 – 2.42) |  |
| **Age group** | | | |
| 18 – 44 years | 1 | 1 | <0.001 |
| 45 – 64 years | 0.93 (0.89 – 0.96) | 0.85 (0.79 – 0.92) |  |
| 65 – 74 years | 0.90 (0.86 – 0.95) | 0.75 (0.69 – 0.80) |  |
| ≥75 years | 0.87 (0.83 – 0.91) | 0.70 (0.65 – 0.75) |  |
| **Sex** | | | |
| Male | 1 | 1 | <0.001 |
| Female | 0.91 (0.88 – 0.93) | 0.90 (0.86 – 0.94) |  |
| **Country of birth** |  |  |  |
| Australia | 1 | 1 | <0.001 |
| Other | 1.09 (1.06 – 1.11) | 1.10 (1.05 – 1.15) |  |
| **Rurality** | | | |
| Urban | 1 | 1 | <0.001 |
| Rural | 0.98 (0.96 – 1.00) | 1.14 (1.09 – 1.18) |  |
| **Charlson comorbidity (excluding malignancy)** | | | |
| No | 1 | 1 | <0.001 |
| Yes | 1.06 (1.03 – 1.10) | 1.40 (1.35 – 1.48) |  |
| **Mental health disorders** | | | |
| No | 1 | 1 | <0.001 |
| Yes | 1.10 (1.03 – 1.16) | 1.62 (1.55 – 1.73) |  |
| **Socio economic status** | | | |
| Q1 (Most disadvantaged) | 1 | 1 | <0.001 |
| Q2 | 1.01 (0.98 – 1.05) | 0.95 (0.91 – 1.02) |  |
| Q3 | 0.96 (0.92– 0.99) | 0.83 (0.76 – 0.89) |  |
| Q4 | 0.93 (0.90 – 0.97) | 0.79 (0.67 – 0.83) |  |
| Q5 (Most advantaged) | 0.82 (0.79 – 0.85) | 0.67 (0.62 – 0.72) |  |
| **COVID-19 lockdown** | | | |
| No | 1 | 1 | <0.001 |
| Yes | 0.95 (0.92 – 0.99) | 0.87 (0.83 – 0.91) |  |
| **Cancer type** | | | |
| Breast | 1 | 1 | <0.001 |
| Digestive organs including colorectal | 1.32 (1.25 – 1.38) | 1.72 (1.59 – 1.84) |  |
| Lung | 1.30 (1.23 – 1.37) | 1.82 (1.67 – 1.98) |  |
| Prostrate | 0.86 (0.81 – 0.92) | 0.91 (0.81 – 1.02) |  |
| Other cancers/ unknown primary site/ill defined | 1.13 (1.10 – 1.16) | 1.38 (1.28 – 1.49) |  |
| **Cancer morphology** | | | |
| Well or moderately differentiated | 1 | 1 | <0.001 |
| Poorly or undifferentiated | 1.09 (1.04 – 1.13) | 1.06 (1.00 – 1.10) |  |
| Unspecified | 1.03 (1.00 – 1.05) | 1.11 (1.05 – 1.16) |  |
| **Pre-index outpatient use** | | | |
| No use /low use | 1 | 1 | <0.001 |
| High use (≥3/year) | 0.99 (0.96 – 1.00) | 1.22 (1.16 – 1.30) |  |
